# Hydrocéphalie du nouveau-né et du nourrisson au Centre Hospitalier National de Nouakchott

**DOI:** 10.11604/pamj.2020.36.184.18750

**Published:** 2020-07-14

**Authors:** Sidi Salem-Memou, Sidiya Chavey, Hamdy Elmoustapha, Abdallahi Mamoune, Ahmedou Moctar, Sidimohamed Salihy, Najat Boukhrissi

**Affiliations:** 1 Service de Neurochirurgie, Centre Hospitalier National, BP 612, Nouakchott, Mauritanie,; 2 Service de Pédiatrie, Centre Hospitalier National, BP 612, Nouakchott, Mauritanie

**Keywords:** Hydrocéphalie, nourrisson, Mauritanie, Hydrocephalus, infant, Mauritania

## Abstract

L´hydrocéphalie du nouveau-né et du nourrisson constitue un facteur important de mortalité et de morbidité dans les pays en voie de développement avec des moyens diagnostiques et thérapeutiques limités. Cette étude avait pour objectif de rapporter notre expérience dans la prise en charge de cette pathologie en Mauritanie. Il s´agissait d´une étude rétrospective portant sur 126 dossiers d´enfants d´âge compris entre 0 et 24 mois, pris en charge pour hydrocéphalie dans le service de neurochirurgie du CHN de Nouakchott, de juin 2014 au juin 2018. Le délai moyen de suivi était de 15 mois (9-27 mois). L´âge moyen au diagnostic était de 5 mois (2 jours à 20 mois). On notait une prédominance féminine (sex-ratio de 0,77). La série était composée de 45 nouveau-nés (35,7%) et de 81 nourrissons (64,3%). Un antécédent infectieux de la grossesse avait été retrouvé dans 19,8% et une infection néonatale dans 23,8%. Sur le plan clinique, 87,3% avaient une macrocéphalie, 35,7% un retard du développement psychomoteur et 15,8% un refus de téter. La principale étiologie était représentée par les myéloméningoceles (23,8%), suivie de la méningite (15,8%). La dérivation ventriculo-péritonéale (DVP) était le principal traitement chez le nouveau-né (68,8%), tandis que la ventriculocisternostomie endoscopique (VCE) était préférée chez le nourrisson (74,1%). Le taux global de complications était de 26,1% (57,6% pour les DVP et 4,1% pour la VCE). L´hydrocéphalie est la principale pathologie de la neurochirurgie pédiatrique en Afrique. Sa prise en charge reste tardive, d´où l´importance de la prévention surtout des anomalies du tube neural et des infections.

## Abstract (en)

Neonatal and infant hydrocephalus is an important factor for mortality and morbidity in developing countries with limited diagnostic and therapeutic means. The purpose of this study was to report our experience in the management of this disease in Mauritania. We conducted a retrospective study of 126 medical records of newborns aged 0-24 months treated for hydrocephalus in the Department of Neurosurgery at the Nouakchott National Hospital from June 2014 to June 2018. Mean follow-up time was 15 months (9-27 months). The average age of patients was 5 months (2 days-20 months). Highest prevalence was observed among female babies (sex ratio 0.77). Our case series consisted of 45 newborns (35.7%) and 81 infants (64.3%). A history of infection during pregnancy was found in 19.8% of cases and neonatal infection in 23.8% of cases. Clinically, 87.3% had macrocephalus, 35.7% had psychomotor retardation and 15.8% refused to suckle. The main cause was myelomeningocele (23.8%), followed by meningitis (15.8%). Ventriculo-peritoneal derivation (VPD) was the first-line treatment in newborns (68.8%), while endoscopic ventriculocisternostomy (EDV) was the preferred approach in infants (74.1%). Overall complication rate was 26.1% (57.6% for VPD and 4.1% for EDV). Hydrocephalus is the most common disease treated by paediatric neurosurgeons in Africa. Management is usually delayed, hence the importance of prevention, especially of neural tube defects and infections.

## Introduction

L´hydrocéphalie est l´une des pathologies les plus fréquentes en neurochirurgie pédiatrique. Elle représente un facteur important de morbi-mortalité [1]. Sa prévalence et son incidence dans de nombreux pays d´Afrique subsaharienne et notamment en Mauritanie sont inconnus. Sa prise en charge dans ces pays est également le plus souvent retardée due à la sous-médicalisation, la pauvreté, aux croyances socioculturelles et taboues qui entourent cette pathologie [1]. Ce retard est responsable des hydrocéphalies monstrueuses qui font la particularité de cette pathologie dans nos milieux. On estime que moins de 10% des cas sont traités annuellement en Afrique [2]. Nous exposons à travers ce travail les résultats de la prise en charge de l´hydrocéphalie du nouveau-né et du nourrisson en Mauritanie tout en incitant sur les difficultés et les défis auxquels nous sommes confrontés.

## Méthodes

Il s´agissait d´une étude rétrospective portant sur 126 enfants âgés de 0 à 24 mois hospitalisés et traités d´une hydrocéphalie dans le service de neurochirurgie du centre hospitalier national de Nouakchott, unique service de neurochirurgie du pays sur une période de 4 ans, de 2014 à 2018. Les enfants atteints d´hydrocéphalie externe et a vacuo ont été exclus. Les informations étaient recueillies à partir des registres et des dossiers médicaux des patients. Les paramètres étudiés étaient : épidémiologie, clinique, paracliniques, thérapeutiques et pronostiques. Le délai moyen de suivi était de 15 mois (9-27 mois).

## Résultats

L´hydrocéphalie du nourrisson représentait 46,3% des hydrocéphalies traitées dans le service durant la période d´étude. La fréquence annuelle moyenne était de 31,5 cas. On notait une prédominance féminine (sex-ratio de 0.77). La série était composée de 45 nouveau-nés (35.7%) et de 81 nourrissons (64.3%). L´âge moyen au diagnostic était de 5 mois avec des extrêmes de 2 jours à 20 mois. La majorité des patients était de bas niveau socioéconomique et provenait des zones rurales (80%). On notait un épisode infectieux maternel dans 19,8% au 1er trimestre de la grossesse. Une échographie anténatale avait été réalisée dans 33,3%. La prophylaxie à base d´acide folique était rapportée dans 13,4% des cas. L´accouchement était médicalisé dans 90% dont 35,7% par césarienne. La consanguinité au premier degré était présente dans 7,9%. Parmi les antécédents périnataux on notait : la prématurité (7,9%), la souffrance néonatale (9,5%), l´ictère néonatal (4,7%), l´infection néonatale (23,8%) dont 15,8% était des méningites confirmées. Le diagnostic anténatal de l´hydrocéphalie n´a été fait dans aucun cas. La macrocrânie était apparue secondairement après la naissance dans 50% des cas. Le délai moyen de consultation était de 3 mois (1 jours à 14 mois). La macrocéphalie (87,3%), les vomissements (27,7%), le retard du développement psychomoteur (35,7%), le refus de téter (15,8%) constituaient les principaux motifs de consultation. Le bombement de la fontanelle antérieure (50%), la dilatation des veines épicrâniennes (73%), le regard « en coucher de soleil » (35,7%), l´hypotonie (7,6%), strabisme (10,3%), l´état général altéré avec troubles de la conscience (11,9%) étaient les signes retrouvés à l´examen physique. Les signes cliniques sont résumés dans le Tableau 1. Le Tableau 2 résume la répartition des patients selon les périmètres crâniens.

**Tableau 1:** les principaux signes cliniques retrouvés dans notre série

Variable	Effectif	Pourcentage (%)
Macrocéphalie	110	87,3
Bombement de la fontanelle antérieure	63	50
Regard «en coucher de soleil»	45	35,7
Dilatation des veines épicrâniennes	92	73,01
Vomissements	35	27,7
Cris incessants et refus de téter	20	15,8
Retard du développement psychomoteur	45	35,7
Hypotonie	20	35,7
Strabisme	13	10,3
Troubles de la conscience	15	11,9
Convulsion	5	3,9
Diminution des réflexes archaïques	10	7,9
Nystagmus	5	3,9
Déficit sensitivomoteur des membres inférieurs	20	15,8

**Tableau 2:** répartition des patients selon le périmètre crânien

Variable	Effectif	Pourcentage (%)
+ 3 DS	77	61,1
+ 2 DS	33	26,2
+ 1 DS	7	5,5
Normal	9	7,1
Total	126	100

DS: Dérivation standard

Le diagnostic positif de l´hydrocéphalie était fait principalement par le scanner cérébral (84,1%), et L´échographie transfontannellaire (15,9%). L´imagerie par résonance magnétique complémentaire était réalisée dans 63,5%. L´étiologie de l´hydrocéphalie était représentée par les myéloméningocèles (23,8%), la méningite (15,8%), les hémorragies intraventriculaires (11,9%), la sténose de l´aqueduc de Sylvius (11,9%), la malformation de Dandy-Walker (13,4%), les tumeurs de la fosse postérieure (3,9%). Dans 19% l´étiologie n´était pas retrouvée (Tableau 3). Sur le plan thérapeutique, la dérivation ventriculo-péritonéale (DVP) était réalisée dans la majorité des cas d´hydrocéphalie chez le nouveau-né 68,8%, tandis que la ventriculocisternostomie endoscopique (VCE) étaient le premier choix pour les hydrocéphalies du nourrisson 74,1%. La dérivation ventriculaire externe était réalisée transitoirement pour les cas d´infection postopératoire. Les valves utilisées étaient de moyenne pression et non réglables. Le suivi postopératoire était en moyenne de 15 mois (9-27 mois). Les complications postopératoires étaient dominées par les infections (15,8%) dont 6 méningites, 4 infections du trajet sous cutané avec extériorisation du cathéter distal à travers la fistule cutanée et 5 infections cutanées retro-auriculaires avec exposition du corps de valve. Les complications mécaniques étaient dominées par les complications hydrodynamiques dans 7 cas (5,5%), suivies par les déconnections dans 03 cas (2,3%). Les complications de la ventriculocisternostomie endoscopique étaient représentées par les fuites transitoires de LCR (2,3%). Le taux global de complications était de 26,1% (57,6% pour les DVP et 4,1% pour la VCE). La durée moyenne d´hospitalisation pour les cas non compliqués était de 4 jours (2-10jours) et pour les cas compliqués de 30 jours (13-55 jours). Le taux de succès pour la VCE était de 94,5%. Les révisions de valve avaient concernés 15 cas soit 28,8%. La mortalité globale était de 4,7%. Deux enfants sont décédés d´une complication du traitement de l´hydrocéphalie.

**Tableau 3:** les principales étiologies de l´hydrocéphalie retrouvées dans notre série

Variable	Effectif (n)	Pourcentage (%)
Myéloméningoceles	30	23,8
Méningite	20	15,8%
Hémorragies intraventriculaires	15	11,9
Sténose de l´aqueduc de Sylvius	15	11,9
Malformation de Dandy-Walker	17	13,4
Tumeurs de la fosse postérieure	5	3,9
Indéterminées	24	19,04

## Discussion

L´hydrocéphalie se définit comme un trouble de l´hydrodynamique du liquide cérébrospinal (LCS) dont la résultante est une dilatation active des cavités ventriculaires. Elle est à l´ origine d´une macrocrânie chez le nouveau-né et le nourrisson [3]. L´hydrocéphalie est connue depuis l´antiquité. Le premier cas a été signalé par Cooke en 1811[4]. En Afrique, Clifford a publié le premier article sur l´hydrocéphalie en 1963 [5] et 20 ans plus tard, Peacock et Currer ont publiés d´importantes séries de cas sur l´hydrocéphalie [6]. Moins de 1% des articles sur l´hydrocéphalie, indexés sur Pubmed sont liés à l´Afrique [7]. L´hydrocéphalie de l´enfant constitue un problème sanitaire pour les pays en voie de développement; notamment africains où 90% des enfants atteints ne seraient pas traités [2,7]. Il s´agit d´une affection grave, pouvant compromettre le pronostic vital ou fonctionnel en l´absence de prise en charge thérapeutique correcte et précoce. Dans les pays développés, l´incidence de l´hydrocéphalie congénitale est de 0,5 à 1/1000 naissances vivantes, tandis que celle de l´hydrocéphalie acquise néonatale est de 3-5/1000 naissances vivantes [8-11]. Bien que l´hydrocéphalie soit plus répandue dans les pays en développement, sa prévalence reste à déterminer [12,13]. L´incidence actuelle de l´hydrocéphalie en Afrique subsaharienne est inconnue. La majorité des enfants atteints d´hydrocéphalie en Afrique sub-saharienne n´accède pas aux structures sanitaires dédiées au traitement de cette pathologie, en raison de la pauvreté [1]. De plus, il y a une distribution inégale des ressources neurochirurgicales à travers le continent, avec 86% des neurochirurgiens en exercice situés en Afrique du Sud et en Afrique du Nord [14]. En Afrique saharienne, il y a environ 1 neurochirurgien pour 5 millions d´habitants et en Afrique de l´Est 1 neurochirurgien pour 10 millions [15,16], contre 1 neurochirurgien pour 100 000 habitants dans les pays européens [14].

En Mauritanie, pour une population de 4 millions d´habitants, il existe un seul service de neurochirurgie avec 6 neurochirurgiens en exercice, soit un neurochirurgien pour 660000 habitants. En utilisant les valeurs de l´incidence de l´hydrocéphalie dans les pays développés et avec un taux brut de natalité qui s´élève à 32%, la Mauritanie devrait compter pas moins de 100 cas hydrocéphalie congénitale par an et 400 cas d´hydrocéphalie néonatale par an. En comparent ces chiffres avec les 126 cas d´hydrocéphalie du nouveau-né et du nourrisson traitées dans notre service sur une période de 4 ans, on réalise bien l´ampleur de l´insuffisance de la prise en charge de cette pathologie dans notre pays. Une combinaison de facteurs médicaux, socio-économiques et culturels sont à l´origine de cette insuffisance de prise en charge : tels que : la honte de porter une enfant malade, l´ignorance du traitement, la pauvreté, les tentatives de traitements traditionnels, une mauvaise compréhension des signes et symptômes précoces, les tabous sociaux et l´accès limité au ressources déjà limitées en matière de santé. Dans ce contexte, les patients qui arrivent à notre service présentent le plus souvent des formes d´hydrocéphalie très évoluées, avec pour plus de 70% d´entre eux un PC supérieur à +3 dérivations standards (Tableau 2) et un important retard du développement psychomoteur. Ces formes évoluées peuvent être évitées par un diagnostic et une prise en charge précoces. Bien que les méthodes de choix pour l´étude de l´hydrocéphalie soient l´IRM et la TDM [17-19], l´échographie trans-fontanellaire joue un rôle important dans le diagnostic et la caractérisation des lésions cérébrales chez le nouveau-né. Elle est considérée comme une méthode de choix pour l´évaluation du nouveau-né à risque, étant même, dans la plupart des cas, la seule méthode nécessaire [20]. En Mauritanie, l´ETF n´est pas utilisée de manière routinière. Elle a permis de confirmer le diagnostic de l´hydrocéphalie dans seulement 15,9% des cas de notre série. Etant plus disponible que le scanner et l´IRM notamment dans les zones rurales éloignées, relativement facile à réaliser et permet aisément de faire le diagnostic positif de hydrocéphalie ; l´ETF doit être vulgarisée dans nos centres de santé et le personnel soignant doit être formé et habilité à la pratiquer. La vulgarisation de sa pratique pourrait avoir un impact positif sur le délai du diagnostic et donc sur le pronostic de l´hydrocéphalie.

La pathogenèse de l´hydrocéphalie pédiatrique est multiple et son étiologie diffère selon le niveau de développement du pays. Chez les nourrissons, l´hydrocéphalie sans cause extrinsèque évidente est généralement appelée hydrocéphalie congénitale, car elle est souvent présente à la naissance. Lorsque l´hydrocéphalie survient en tant que complication d´une autre affection telle qu´une hémorragie, une infection ou un néoplasme, elle est généralement appelée hydrocéphalie acquise ou secondaire [10]. Dans les pays développés L´hydrocéphalie pédiatrique résulte généralement d´une hémorragie intraventriculaire de la prématurité ou de causes congénitales. En Afrique, l´étiologie post-infectieuse varie de 7 à 60% et reste corrélée au niveau de santé de la population du pays [6,13,21]. Elle constitue la principale cause de l´hydrocéphalie du nouveau-né et du nourrisson, en raison d´une plus grande incidence de septicémie néonatale. Cependant, cette prédominance des hydrocéphalies post-infectieuses à tendance à s´atténuer, surtout chez le nourrisson avec un profil étiologique qui tend vers celui des pays développés [21]. Cette tendance se confirme dans notre étude avec seulement 15,8% des cas pour l´étiologie post-infectieuse contre 49,2% pour les causes malformatives (Tableau 3). Les malformations du tube neural étaient l´étiologie malformative la plus fréquente dans notre série (23,8%). Si la survenue de ces malformations est d´origine multifactorielle (facteurs génétiques, environnementaux...), il est cependant établi qu´elle est corrélée à des apports faibles en acide folique (ou vitamine B9). Malgré les efforts entrepris dans le domaine de la prévention par la généralisation des programmes élargis de vaccination, Beaucoup reste cependant à faire dans la sensibilisation des populations. Un meilleur accès des femmes en âge de procréer à une supplémentation prénatale en acide folique pourra réduire d´avantage la fréquence de cette pathologie aux lourdes séquelles neurologiques.

La dérivation ventriculo-péritonéale reste le traitement de choix de l´hydrocéphalie. Les taux de complications varient entre 1 et 50%, deux ans après la chirurgie [2,12,22-25]. Dans les séries de l´Afrique subsaharienne, les complications décrites varient entre 7% et 69% et sont liées à des causes mécaniques (11-54%) et à l´infection (7-69%) [6,13,23,24,26-28]. La pose de DVP est plus dangereuse dans le contexte africain que dans les pays développés en raison des complications infectieuses et du dysfonctionnement de valve [25]. Choux et al rapportent que la mise en œuvre de protocoles et la formation du personnel aux mesures de contrôle des infections liées aux périodes préopératoire, opératoire et postopératoire permettent de réduire sensiblement les complications infectieuses [29]. La VCE est actuellement utilisée dans un large éventail d´indications dans le traitement de l´hydrocéphalie. Elle est même utilisée dans les hydrocéphalies post-méningitiques qui étaient longtemps considérées comme une contre-indication relative, avec des taux de succès situés entre 59 et 77% [13,30-32]. D´après Warf en Ouganda [33], une VCE associée à une coagulation bilatérale des plexus choroïdes a une plus grande chance de succès dans l´hydrocéphalie post-infectieuse. De plus, la VCE évite les complications liées à l´implantation de matériels et semble particulièrement adaptée dans un contexte de ressources financières limitées grâce aux avantages qu´elle offre en terme de rentabilité et de réduction de la morbidité [34,35]. Dans notre série, son indication tenait compte de l´âge du patient et de l´étiologie de l´hydrocéphalie. Dans les pays en voie de développement, au-delà des infrastructures onéreuses que requièrent la réalisation sécurisée des interventions chirurgicales, la disponibilité des explorations diagnostiques et le suivi hospitalier qui se déroule essentiellement dans les grandes villes, le coût hospitalier du traitement des hydrocéphalies génère des dépenses disproportionnelles par rapport au pouvoir d´achat des familles. Les subventions ou autres exonérations gouvernementales étant rarissimes, le financement des soins hospitaliers d´un enfant hydrocéphale est à l´origine de l´enlisement dans la pauvreté de toute la famille.

## Conclusion

L´hydrocéphalie du nouveau-né et du nourrisson est une pathologie assez fréquente dont les conséquences sont graves. Améliorer la qualité des soins dispensés aux patients atteints d´hydrocéphalie dans un pays à contexte social, économique et culturel tel que la Mauritanie représente un défi qui dépasse de loin l´aspect du traitement neurochirurgical. Malgré les efforts entrepris dans le domaine de l´équipement neurochirurgical et de la formation des neurochirurgiens aux techniques endoscopiques, beaucoup reste cependant à faire dans la sensibilisation des populations et du personnel soignant. Nous insistons sur l´importance de la prévention des anomalies du tube neural et des hydrocéphalies post infectieuses.

### Etat des connaissances sur le sujet

- L´hydrocéphalie du nouveau-né et du nourrisson constitue un facteur important de mortalité et de morbidité en Afrique;
- Il s´agit d´une affection grave, pouvant compromettre le pronostic vital ou fonctionnel en l´absence de prise en charge thérapeutique correcte et précoce;
- En Afrique, l´étiologie post-infectieuse constitue la principale cause de l´hydrocéphalie du nouveau-né et du nourrisson.

### Contribution de notre étude à la connaissance

- Notre étude apporte l´expérience mauritanienne dans la prise en charge de l´hydrocéphalie du nouveau-né et du nourrisson;
- Elle soutient la diminution de l´étiologie post-infectieuse déjà constatée dans d´autres régions d´Afrique.
